# Palladium: a future key player in the nanomedical field?

**DOI:** 10.1039/c5sc00070j

**Published:** 2015-01-19

**Authors:** Anaëlle Dumas, Patrick Couvreur

**Affiliations:** a Institut Galien Paris-Sud , Université Paris-Sud , UMR CNRS 8612 , Faculté de Pharmacie , 5 rue Jean-Baptiste Clément , 92296 Châtenay-Malabry Cedex , France . Email: patrick.couvreur@u-psud.fr

## Abstract

Metal nanostructures offer invaluable possibilities for targeted drug delivery, detection/diagnosis and imaging. Whereas iron, gold, silver and platinum nanoarchitectures have largely dominated this field to date, several hurdles impede the widespread application of those nanopharmaceuticals in a clinical context. Therefore, technologies based on alternative metals are now being evaluated for their potential in medical applications. Palladium nanostructures are characterized by remarkable catalytic and optical properties. However, until recently, very few studies have taken advantage of these unique characteristics for applications in the biomedical field. Very recently, palladium nanostructures have been reported as prodrug activator, as photothermal agents and for anti-cancer/anti-microbial therapy. With only a handful of reports available, the pharmaceutical applications of palladium nanostructures reviewed here are in their infancy. Yet their interesting performance and toxicity profiles may qualify them as future key players in the nanomedical field.

## Abstract 2

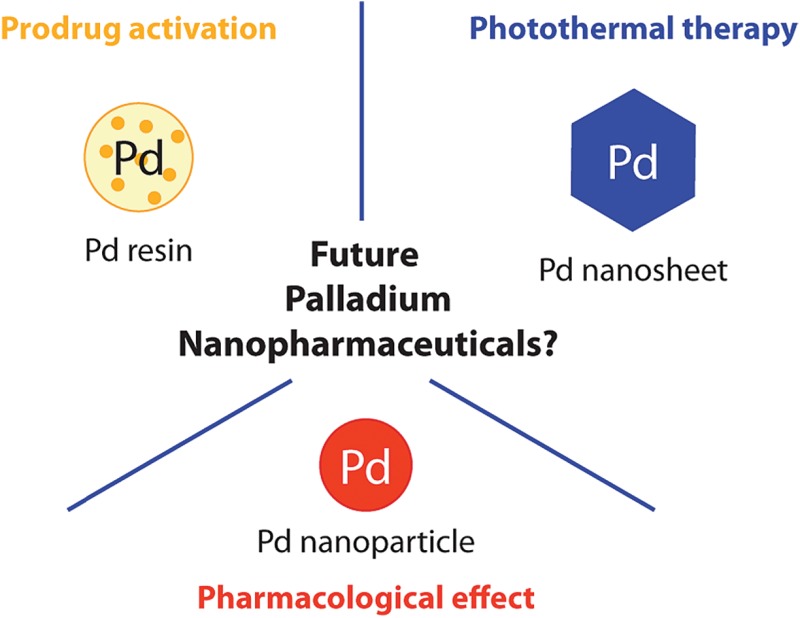
Palladium nanostructures with therapeutic potential are emerging as innovative tools in the nanomedical field.

## Context

The introduction of nanotechnologies in the biomedical field has empowered a myriad of applications for targeted drug delivery, detection/diagnosis and imaging, and are on the way to positively impact medicine.^1–3^ Due to their size, structure versatility and optoelectronic properties, metal nanostructures are attracting interest for a number of biomedical applications.^4–6^ Indeed, advances in their synthesis and surface engineering by specific ligands enable the preparation of nanostructures with controlled size, shape, dispersity, and in some cases biocompatibility, which has allowed the development of an increasing number of drug-like metal architectures.^7^ Iron, gold, silver and platinum nanostructures are the most extensively studied examples of metal nanodrugs with some preparations already subject to clinical trials or even already marketed.^4,5^ However, the highly complex, multifaceted and multifactorial interactions of metal nanoparticles with biological systems, and the absence of long term toxicological data, impede the widespread application of those nanopharmaceuticals.^8,9^ While continuous efforts are made to improve their activity and safety profiles for translation to the clinic, technologies based on alternative metals are evaluated in parallel, for their potential in medical applications. Palladium is a noble metal with remarkable catalytic, mechanic and electronic properties. Palladium nanostructures have gained interest in the last decade in a number of applications including catalysis (*e.g.*: hydrogenation, C–C bond formation and oxidation, for the manufacture of pharmaceuticals, the processing of environmental pollutants or automotive emission control),^10–13^ composition of electrical equipment,^14,15^ or as sensors for the detection of various analytes.^16–19^ In medicine, palladium is nowadays commonly used in dental appliances^20^ and ^103^Pd needles are used in the clinic for prostate cancer^21^ and choroidal melanoma brachytherapy.^22^ However, despite their remarkable properties, palladium nanostructures have not been exploited, until recently, in the nanomedicine field and it is the aim of this Minireview to highlight such potential applications.

**Fig. 1:**
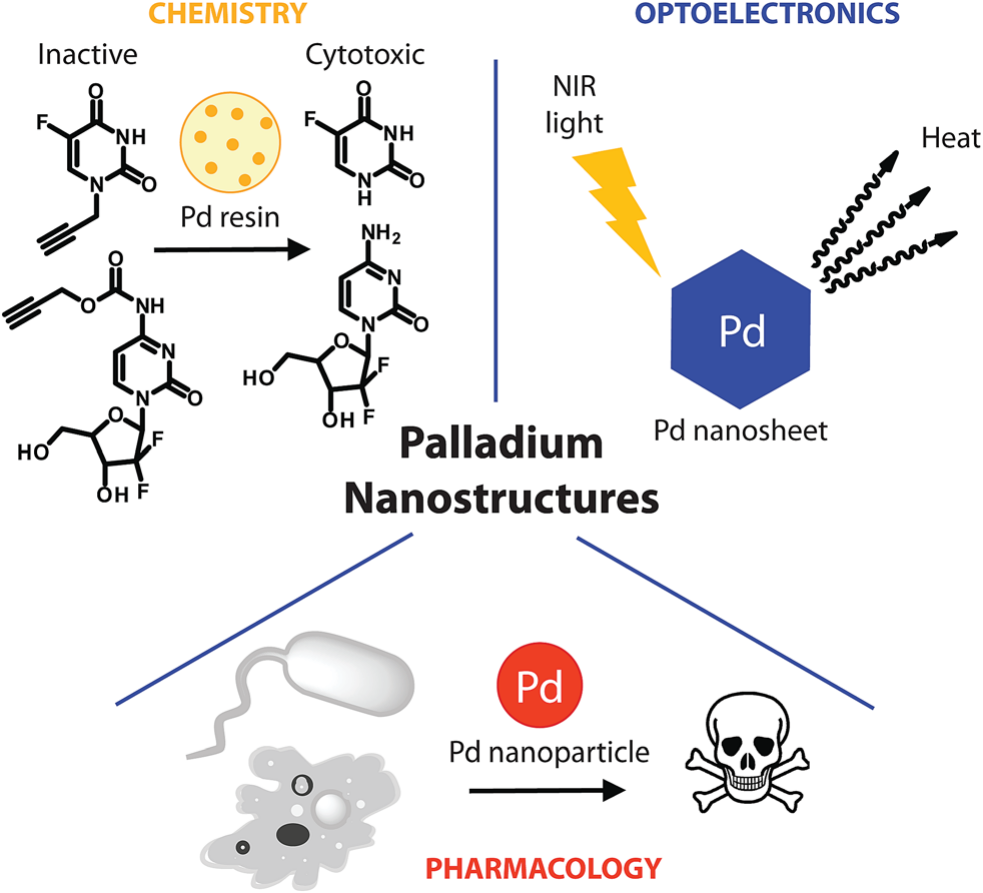
Schematic representation of palladium nanopharmaceuticals: Pd(0)-resin for the chemical activation of prodrugs, Pd nanosheets for the photothermal conversion of NIR light and Pd nanoparticles as anti-microbial and cytotoxic agents.

Taking advantage of the metal catalytic and/or optical properties, potential therapeutic applications have been considered through three classes of Pd nanostructures (Fig. 1). Particularly remarkable is the nanotechnologies-mediated chemistry, involving palladium resins to trigger therapeutically relevant chemical reactions in a biological setting. This approach enables spatially-targeted chemical activation of prodrugs and has no precedent among the therapeutic strategies offered by metal nanostructures. In other recent reports, Pd nanosheets and Pd nanoparticles (NPs) were used, respectively, for photothermal therapy and as anti-tumor/anti-microbial agents. As detailed below, such Pd-based nanopharmaceuticals could become part of the nanomedical repertoire soon.

## Palladium nanostructures of therapeutic potential

### Pd(0)-functionalized resins for targeted prodrug activation

Chemistry offers a unique means to specifically manipulate biological systems. To date, a large repertoire of bioorthogonal reactions enables the specific formation or cleavage of chemical bonds for the modification of molecules in the chemically complex environment of biological systems.^23,24^ A number of these reactions have demonstrated utility for the specific labelling, tracking, activation and manipulation of molecules under physiological conditions. However, metabolic or genetic engineering is often required for the introduction of a selective reactive chemical handle in a biological milieu.^25^ Added to toxicological issues and selectivity concerns associated with some of these reactions, their use has generally been restricted to mechanistic studies and/or to the dissection of cellular processes, rather than therapeutic applications.^26^

Recently, a heterogeneous Pd(0)-mediated reaction for the spatially-targeted activation of prodrugs was reported. Using a polyethylene glycol–polystyrene resin supporting 5 nm Pd(0) particles (Pd(0)-resin), Unciti-Broceta *et al.* demonstrated the possibility to bioorthogonally activate 5-fluoro-1-propargyluracil,^27^ and *N* ^4^-propargyloxycarbonylgemcitabine,^28^ two otherwise biologically inert prodrugs of, respectively, 5-fluorouracil and gemcitabine. Although independently harmless, combined treatment of either of the prodrugs with Pd(0)-resin restored cytotoxicity and led to equivalent anti-proliferative properties compared to the unmodified drug in colorectal and pancreatic cancer cells. Heterogeneous deprotection of the propargyl group using immobilized palladium nanoparticles enabled control over the reaction location, which could not be achieved with the most commonly used bioorthogonal reactions, due to their homogenous character. Intratumoral implants loaded with Pd nanoparticles could enable spatially-controlled activation of otherwise highly toxic compounds. This could significantly expand the therapeutic window of potent cytotoxic drugs, whose clinical activity is currently limited by their safety profile. Further *in vivo* demonstration will, however, be crucial to evaluate the real therapeutic potential of this approach, in terms of safety and pharmacological efficiency. Nevertheless, preliminary studies indicated that Pd(0)-resin beads introduced in the yolk sac of zebra fish embryos displayed neither apparent toxicity nor any development alteration over several days, while retaining their chemical activity.^27^

Nanoparticles mediating chemical reactions of therapeutic interest in a biological environment represent a new approach, beyond the state-of-the-art, to targeted drug-delivery. Chemically-controlled drug release using palladium resin could be applied to a number of active compounds and diseases as long as a palladium-labile prodrug can be prepared that is: (i) minimally toxic, (ii) highly sensitive to palladium and (iii) chemically stable towards enzymatic processing.^29^

### Pd nanosheets for cancer photothermal therapy

Metal nanostructures-mediated photothermal (PT) therapy has attracted extensive interest in recent years. This technology involves the absorption of photon energy by the tumor localized nanostructures, which convert it to heat, causing death of surrounding cells.^30^ For this purpose, the use of a near-infrared (NIR) light source is generally needed for deep tissue penetration, while causing little damage to healthy cells. Gold nanostructures are the most widely studied PT therapy agents due to their high extinction coefficients in the NIR region, high PT conversion efficiency and acceptable biocompatibility.^31,32^ However, these structures usually suffer from low thermal stability, leading to rapid deactivation under the heat generated upon irradiation.^33,34^

Palladium nanostructures were not considered as efficient PT agents until Huang *et al.* reported the synthesis of ultra-thin (1.8 nm) hexagonal Pd nanosheets, displaying well-defined, size-dependent and tunable absorption peaks in the NIR region with efficient PT conversion.^33^ The 41 nm edge nanosheets were able to kill 100% liver cancer cells after 5 min irradiation with an 808-nm laser, while showing high biocompatibility in the absence of irradiation. Interestingly, these nanosheets show better photostability when compared to gold or silver nanostructures. Several modifications including surface coating and functionalization have then been proposed to improve properties of Pd nanosheets such as cellular uptake, or PT conversion efficiency.^35,36^ Porous Pd nanoparticles (22.8 nm) were also recently reported as attractive PT agents with PT conversion efficiency as high as 93.4%, which is comparable to typical Au nanorods.^37^

Of particular interest for biomedical applications was the development of smaller reduced-glutathione (GSH)-coated palladium nanosheets (<10 nm diameter) with demonstrated renal clearance.^38^ In the absence of irradiation, these GSH-coated ultra-small Pd nanosheets showed prolonged blood circulation, accumulation in tumors and no apparent toxicity after injection in mice, whereas irradiation using a 808-nm NIR laser (at 1 W cm^–2^ for 5 min) enabled complete tumor ablation *in vivo*.^38^ In addition, due to their size sitting below the renal filtration limit, renal clearance was observed, making these ultra-small Pd nanosheets promising for practical use in PT cancer therapy.

The attractive properties of these nanodevices as PT agents motivated further use as components of more complex assemblies for combined PT-chemo- or PT-photodynamic therapies. Typically, doxorubicin (Dox)-loaded mesoporous silica NPs covered with the Pd nanosheets provided synergistic combined PT and chemo treatment: the heat resulting from NIR light conversion promoted the pH-dependent release of Dox after endocytosis, and the cellular uptake of the Pd nanosheets was improved by their loading on the silica nanospheres.^39^ A similar therapy combination was shown using Pd@Ag nanoplate composites.^40^ Interestingly, photothermally enhanced photodynamic (PD) therapy was also reported by using Pd-based nanodevices. PD therapy consists in the absorption of photons by a photosensitizer resulting in the formation of reactive oxygen species, causing irreversible damage to surrounding cells. For such purpose, Shi *et al.* reported mesoporous silica-coated Pd@Ag nanoparticles covalently loaded with the Chlorin e6 photosensitizer.^41^ After intratumoral injection of these particles in tumor bearing-mice, simultaneous irradiation with 660-nm (Chlorin e6) and 808-nm (Pd nanosheets) lasers completely destroyed the tumor, while irradiation with 660 or 808 nm alone did not.^41^ Further elaboration of this system was reported by Zhao *et al.* with mesoporous silica nanoparticles loaded with tetra-substituted carboxyl aluminium phthalocyanine as a photosensitizer, which allowed efficient PD and PT effects under a single 660-nm laser irradiation.^42^ It should be noted that combined phototherapies requiring a single laser beam provide a significant practical advantage for clinical applications, as the focus of two lasers beams on a single point in a patient is delicate.

Generally, Pd nanostructures offer better photostability and require lower laser power densities compared to other metallic PT therapy agents.^37^ Taken together, high photothermal conversion efficiency, biocompatibility, favorable size and applications in combined therapies make Pd nanoparticles very attractive alternatives to existing agents for non-invasive PT cancer treatment.

### Pd nanoparticles as anti-microbial and cytotoxic agents

Palladium nanostructures have also emerged as self-therapeutics. A few examples recently demonstrated their anti-microbial and cytotoxic pharmacological activities.

For example, Adams *et al.* reported the size dependent high anti-microbial activity of Pd NPs. The particles showed higher growth inhibition against *S. aureus*, compared to *E. Coli*, highlighting Pd NPs as useful anti-microbial agents especially for gram positive bacteria.^43^

Balbin *et al.* reported that Pd NPs supported on mesoporous silica-based materials displayed relatively high cytotoxic activity against four tested human cancer cell lines.^44^ Interestingly, these hybrid materials also displayed catalytic activity and were used for C–C bond formation *via* Suzuki–Miyaura cross-coupling between small molecules.^44^ Even if Pd NPs are interesting candidates as self-therapeutics, further investigations are needed to elucidate their mechanism of action before consideration as relevant nanomedicines.

## Outlook

Despite their advantageous chemical, mechanical and physical properties, palladium nanostructures have long been absent from the nanotherapeutics repertoire. The three approaches to palladium nanomedicines highlighted in this Minireview uncover the therapeutic potential of these structures by the exploitation of their catalytic ability, photothermal efficiency and/or biological activity. Although further *in vivo* evaluation will be crucial, these reports reveal the potential of palladium species to perform similarly or even better than existing metallic nanostructures for the same task.

Crucial to the establishment of palladium nanostructures in the biomedical field will be the determination of their safety profile. This will require the acquisition of a precise understanding of parameters such as: (i) pharmacokinetics and biodistribution, (ii) metabolism and clearance mechanism, (iii) acute and chronic toxicity and (iv) mechanisms behind the pharmacological activity. Interactions of the nanostructures with cells and biological environments (and their impact on pharmacokinetics and biodistribution) are other issues deserving deep investigation, especially after nanoparticle surface modification.^8^

Of note, owing to its biological inertness, metallic Pd(0), forming the Pd nanostructures, shows *a priori* the safest toxicity profile among palladium species.^45^ However, the release of Pd^2+^ ions from Pd(0) structures under biological conditions, especially from catalytic resins undergoing Pd(0)/Pd^2+^ cycles, cannot be excluded and will need careful consideration.

Even if not as nanoparticles, the safety of metallic palladium has already been demonstrated in the clinic where it is commonly used in the form of radioactive ^103^Pd needles injected locally for cancer treatment,^21,22^ or as alloys component in dental appliances.^20^ Although sensitization and allergy can occur in some patients, these technologies are generally considered as safe and not showing palladium-related complications.^45,46^ However, although bulk palladium itself seems relatively harmless, different biodistribution and accumulation patterns, eventually leading to new toxicities, could result from its administration as nanometric structures. Even within chemically similar nanometric objects, small changes in size, shape, composition or surface functionalization can significantly impact the pharmacological activity, the pharmacokinetics and the interactions with biological systems.^8^ This is, however, a burden encountered in the development of all types of nanomedicines since poor predictability requires careful safety assessment on a case by case basis. On the other hand, innovation in the technologies of metallic NP preparation tend to give access to Pd nanostructures with increasingly controlled size, shape and coating, to help the design of fine-tuned structures meeting both therapeutic and safety requirements.^38,47,48^

The emergence of palladium nanotechnologies with potential therapeutic properties is recent, which may explain the surprisingly low number of publications. Yet, their unique properties and potentially low toxicity qualify them as possible future key players in the nanomedical field. There is no doubt that new applications will be discovered, for example by the use of their optical properties for diagnosis or highly attractive theranostic purposes. The remarkable multiple facets of palladium nanostructures might, therefore, soon provide highly simple systems but capable of combined effects.
